# A New Visual Trap for *Rhagoletis cerasi* (L.) (Diptera: Tephritidae)

**DOI:** 10.3390/insects5030564

**Published:** 2014-07-18

**Authors:** Claudia Daniel, Samuel Mathis, Georg Feichtinger

**Affiliations:** 1 Research Institute of Organic Agriculture (FiBL), Ackerstrasse 113, Postfach 219, CH-5070 Frick, Switzerland; 2 Fachstelle Pflanzenschutz Strickhof, Eschikon, Postfach, CH-8315 Lindau, Switzerland; E-Mails: info@kcb-samen.ch (S.M.); georg.feichtinger@strickhof.ch (G.F.)

**Keywords:** *Rhagoletis cerasi*, Diptera, Tephritidae, yellow sticky trap, monitoring

## Abstract

The European cherry fruit fly, *Rhagoletis cerasi* (L.) (Diptera: Tephritidae), is the most important pest of sweet cherries in Europe. The aim of our experiments was to develop a new, cost-efficient, lead chromate-free and more eco-friendly trap for monitoring and mass trapping of *R. cerasi*. Five different-colored yellow panels and three different trap shapes were compared to a standard Rebell^®^ amarillo trap in three experimental orchards in 2012. Trap color F, with a strong increase in reflectance at 500–550 nm and a secondary peak in the UV-region at 300–400 nm, captured significantly more flies than the standard Rebell^®^ amarillo trap. Yellow traps with increased reflectance in the blue region (400–500 nm) were least attractive. Trap shape was of minor importance, as long as the object was three-dimensional and visible from all directions. Based on economic and practical considerations, a cylinder-shaped trap “UFA-Samen Kirschenfliegenfalle” was developed for commercial use and is currently under on-farm evaluation.

## 1. Introduction

The European cherry fruit fly, *Rhagoletis cerasi* (L.) (Diptera: Tephritidae), is the most important pest of sweet cherries *Prunus avium* (L.) L. in Europe. The adult flies emerge from the soil in May and June [1] and begin to lay eggs about ten days after emergence [2,3]. The maggots develop inside the cherries. At the third instar, the larvae leave the fruit, drop to the soil and, within hours, start to pupate under the tree canopy [4]. *R. cerasi* is univoltine and overwinters as a pupa [5]. Without insecticide treatment, up to 100% of fruit can be infested [6]. *R. cerasi* poses a challenge to cherry growers because of the low tolerance level of the fresh market to damaged fruit, with a maximum of 2% of infested fruits. In most orchards and in most years, insecticide treatments are necessary to keep the infestation level below the threshold. The phase-out of organophosphorus insecticides now threatens cherry production throughout the European Union. The use of the systemic insecticide, dimethoate, in particular, is being revoked due to problems with ecotoxicity and residues. Registered alternative products [7] are targeting adult flies. Therefore, the detection of first flight becomes increasingly important to schedule precise insecticide applications. New methods have recently been developed for organic agriculture, such as biocontrol using the entomopathogenic fungus, *Beauveria bassiana* [8], or bait sprays with spinosad or Neem [9,10,11]. These methods also require an exact determination of the first flight. 

*R. cerasi* is known to be highly responsive to visual stimuli [12], especially to yellow surfaces [13,14,15,16,17]. Remund [14] determined that daylight fluorescent yellow-colored flat surfaces are most attractive. Prokopy [18] suggested that large yellow surfaces represent a super-normal foliage-type stimulus, eliciting food-seeking behavior in *R. cerasi*. He also hypothesized that flies react to yellow on the basis of true color discrimination. This hypothesis was supported by Agee *et al.* [19], who showed that adult *R. cerasi* have a major peak of electroretinographically assessed spectral sensitivity at 485 to 500 nm (yellow green region) and a secondary peak at 365 nm (ultraviolet region). Traps with a sharp increase of reflectance in the 500 to 520 nm region were found to be the most attractive to *R. cerasi* [19,20]. Based on this knowledge, the three-dimensional, cross-shaped Rebell^®^ amarillo trap was developed in the 1970s [21] and is now used for monitoring and mass-trapping purposes in Switzerland. In home gardens, mass trapping often is the only available method for cherry fruit fly control, although the efficacy is not always satisfactory. Depending on the tree size, several traps per tree are necessary. In old, standard trees with a canopy diameter of 10 m, approximately 10 traps are needed [14]. At a price of 3.40 € per trap, the Rebell^®^ trap is rather expensive [7]. In addition, the yellow pigments used for the Rebell^®^ trap still contain lead chromate. The toxicity of lead chromate has been known since about 1980 [22], and lead chromate was listed as a “substance of very high concern” (SVHC) in the EU according to the REACH Regulation (Regulation (EC) No 1907/2006) [23]. Rebell® traps can be cleaned and reused, but because cleaning is very time consuming and solvents are necessary to dissolve the glue, most farmers use Rebell® traps only once. The aim of our experiments was to develop an equally attractive, but less material-intensive, lead chromate-free, more eco-friendly and more cost-effective trap for *R. cerasi*.

## 2. Materials and Methods

**Figure 1:**
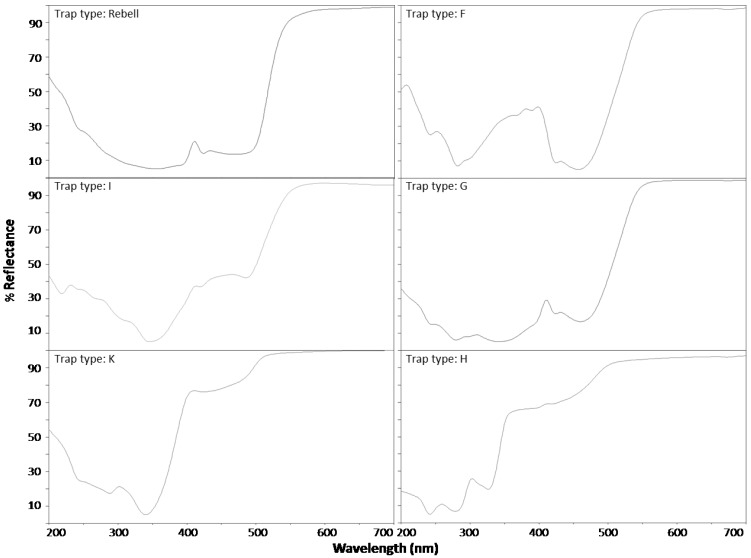
Spectral reflectance curves for the six differently colored panels (without Tangle-Trap) compared in Experiment 1.

Trap types: In order to estimate the performance of the new trap types, two experiments were conducted. In the first experiment, the influence of different shades of yellow was evaluated comparing five differently-colored 15 × 20 cm panels to a Rebell^®^ amarillo (Andermatt Biocontrol, Grossdietwil, Switzerland) panel of the same size. Details on the color and material are given in Table 1 and Figure 1. Polypropylene panels (Schoellkopf, Rümlang Switzerland) were double-printed with yellow (4.2 g color/m^2^) on both panel sides. Polyethylene panels were produced using colored granules (Granula AG, Merenschwand, Switzerland). 

**Figure 2:**
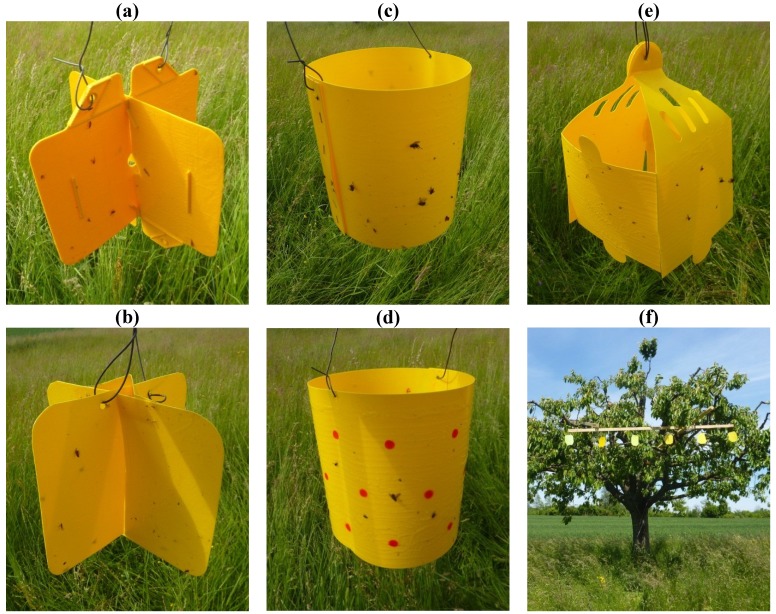
Trap shapes tested in the experiment: (**a**) Rebell^®^ amarillo (cross); (**b**) cross-shaped trap; (**c**) cylinder-shaped trap; (**d**) dotted cylinder-shaped trap; (**e**) cube-shaped trap (the “roof” was not glued); (**f**) one replicate of Experiment 1: a comparison of differently-colored panels.

In the second experiment, the influence of trap shape was evaluated by comparing differently-shaped traps to a standard Rebell^®^ amarillo trap (Table 1, Figure 2). The Rebell^®^ trap consists of two yellow plastic panels fastened together at right angles. This cross-shape was copied with the material and color of Panel I from the color experiment. Additional shapes—a cube with a side length of 15 cm and a cylinder with a height 20 cm and a circumference of 54 cm—were produced from the same material (Figure 2). Furthermore, the combination of yellow surfaces with red dots was evaluated by spraying red dots of 13 mm in diameter in regular distances of 75 mm onto the cylinder-shaped trap. Spray colors of one layer of Fire red” (color code: RAL3000), followed by one layer of “Neon Signalrot fluoreszierend” (Dupli Color, Hassmersheim, Germany), were applied using a stencil. All trap types were glued using Tangle-Trap® (Contec Inc., Victoria, Canada). 

**Table 1:** Material, shape and size of the sticky surface of trap types used in the two experiments.

Trap type	Material (thickness)	Fluorescence	Shape (Sticky surface)	Experiment
Panel Rebell	Rebell^®^ amarillo Polypropylene (1 mm)	No	Panel (600 cm^2^)	Color
Panel I	Polypropylene (0.8 mm)	Yes	Panel (600 cm^2^)	Color
Panel K	Polypropylene (0.5 mm)	Yes	Panel (600 cm^2^)	Color
Panel F	Polyethylene (1 mm)	No	Panel (600 cm^2^)	Color
Panel G	Polyethylene (1 mm)	Yes	Panel (600 cm^2^)	Color
Panel H	Polyethylene (1 mm)	Yes	Panel (600 cm^2^)	Color
Cross Rebell	Rebell^®^ amarillo Polypropylene (1 mm)	No	Cross (1200 cm^2^)	Shape
Cross	Polypropylene (0.8 mm)	Yes	Cross (1200 cm^2^)	Shape
Cube	Polypropylene (0.8 mm)	Yes	Cube (900 cm^2^)	Shape
Cylinder	Polypropylene (0.8 mm)	Yes	Cylinder (1080 cm^2^)	Shape
Dotted Cylinder	Polypropylene (0.8 mm)	Yes	Cylinder + 21 red dots of 13 mm in diameter (1080 cm^2^)	Shape

The spectral reflectance of different colored yellow panels was measured using a Camag TCL 3 scanner. The reflectance and absorption was recorded continuously from 200 to 700 nm and showed clear differences (Figure 1). The standard Rebell^®^ amarillo trap showed a strong increase in reflectance at 500–550 nm. Trap types F and G showed a similar strong increase in the same region. However, trap type F had another pronounced peak of reflectance in the UV region at 300–400 nm, but a very low reflectance at ~450 nm (blue region). Trap types H, I and K had a similar high reflectance above 500 nm, but these traps also showed a high reflectance in the blue region (400–500 nm).

Experimental design: The experiments were conducted in northwestern Switzerland. All three experimental orchards were located in a 3-km radius around Arlesheim BL (47°29'46.20''N; 7°37'01.72''E). The orchards consisted of untreated trees of the varieties Schauenburger, Hedelfinger and Basler Adler. Trees were 30–40 years old, 4–5 m high and had a canopy diameter of 5–6 m. The infestation pressure with *R. cerasi* was high in the previous years. Each trap was tested in three replicates in each orchard for a total of nine replicates. One replicate consisted of one cherry tree: all trap types were randomly fixed on a wooden bar with a distance of 50 cm between traps. The bar was fixed at a 3.5 m height to the south-southeast periphery of each cherry tree (see Figure 2f). This experimental design was chosen in order to test all trap types within the same tree, because infestation pressure can vary greatly, even among neighboring trees. We randomly arranged traps within one replicate and recorded the neighboring trap types in order to statistically correct for any influences between neighboring traps. Experiment 1 and Experiment 2 were conducted simultaneously in the same orchards on separate trees. The first flight of the cherry fruit fly was determined by using an temperature-based forecasting model [24], as well as one Rebell^®^ trap per orchard. These monitoring traps were checked daily, and the experimental traps were installed at the day of first *R. cerasi* catches: on May 15, 2012, in Orchards 1 and 2 and on May 22, 2012, in Orchard 3, which is located at a higher altitude. Traps were removed immediately before cherry harvest on June 19, 2012. 

Examinations: The number of cherry fruit flies per trap was counted in weekly intervals. All insect counts were conducted on the same day in all experimental orchards. Cherry fruit flies were removed from the traps during counting. The fruit load of the experimental trees was estimated (0 = little load; 1 = low load; 2 = normal-high load), as well as the shadow on the experimental traps due to cherry leaves and branches (0 = no shadow on the trap surface from 10 am to 4 pm; 1 = little shadow). Climatic conditions during the experimental period were monitored using a Campbell CR10X meteorological station. 

Statistical analysis: JMP [25] was used for all statistical analyses. If necessary, data were [log10(x + 1)] transformed to obtain a normal distribution. The normality of data and the homogeneity of variance were tested before performing an ANOVA (factors: trap type, orchard, tree, shadow). Means were compared by Tukey’s HSD *post hoc* tests (α = 0.05). Data are presented in the figures and the text as means with standard errors. 

## 3. Results and Discussion

**Figure 3:**
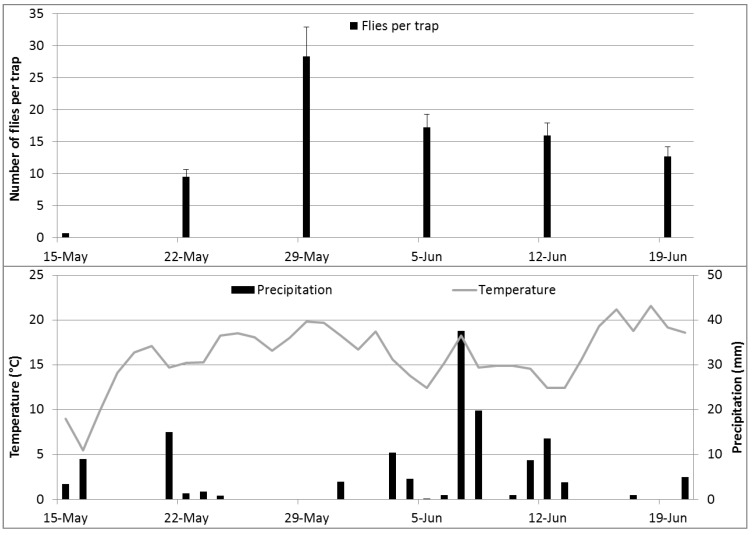
The climatic conditions and cherry fruit fly catches (average over all experimental traps + SE) during the experimental period in 2012.

The climatic conditions and flight activity of cherry fruit flies are given in Figure 3. The highest catches were obtained in the second week after the beginning of the flight period during the warm and sunny period from May 22 to 29, 2012.

A total of 7962 cherry fruit flies were captured during the experimental period (May 15 to June 19, 2012) on the 99 experimental traps. Among the three experimental orchards, significant differences were observed: flight intensity over the whole experimental period was highest in Orchard 1, with 166.5 ± 20.6 flies per trap, followed by Orchard 2, with 68.7 ± 10.8 flies per trap. The lowest flight intensity was observed in Orchard 3 (6.2 ± 1.6 flies per trap; statistical analysis: data transformed [logx + 1]; one-way-ANOVA, F_2,96 _= 69.4, *p* < 0.0001; Tukey’s HSD-test α = 0.05). Within orchards, flight intensity differed among the different experimental trees. However, fruit load on the cherry trees had no influence on trap catches. This might be because the fruit load was very low in most experimental trees following cold and rainy weather conditions during pollination in early spring. Differences between the experimental trees might be the result of the infestation level in the previous year or wind exposition. Due to the experimental design, we expected an influence of neighboring traps, but these influences were very low and statistically not significant. However, shadows from cherry leaves and branches on the experimental traps could not be avoided and influenced catch counts. In order to adjust for influencing factors, we used a four-way-ANOVA for the statistical comparison of trap catches. Included factors were trap type, orchard and tree (nested within orchard) and shadow (yes/no). In order to summarize the results, we analyzed the number of flies per trap captured from May 23 to 29 (main flight activity, beginning of experimental period), from June 13 to 19 (end of experimental period), as well as over the whole experimental period (May 15 to June 19). 

**Figure 4:**
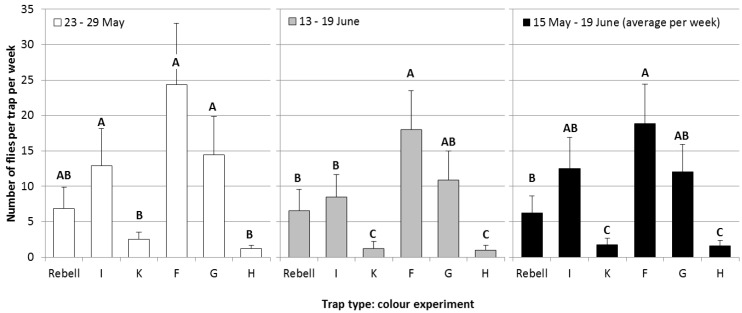
A comparison of differently-colored yellow sticky panels on the numbers of captured cherry fruit flies per week (+SE) during the second week of experiments (white bars), the fifth week of experiments (grey bars) and during the whole experimental period (black bars; average per week; Tukey’s HSD test *p* = 0.05; different letters show significant differences).

Experiment 1, comparison of differently colored panels: The results of the comparison of differently colored panels are given in Figure 4. Over the whole flight period (Figure 4, black bars), trap Type F captured significantly more flies than trap Types K, H and the Rebell^®^ trap, but not more flies than trap Types I and G. Trap Types I and G did not significantly differ from trap Type F or from the Rebell^®^ trap (statistical analysis: data transformed [logx + 1], four-way-ANOVA, Tukey’s HSD-test; May 23–29: F_5,39 _= 8.7, *p* < 0.0001; June 13–19: F_5,39 _= 29.3, *p* < 0.0001; May 15–June 19: F_5,39 _= 32.9, *p* < 0.0001).

The attractiveness of trap Type F is mainly explained by the reflectance pattern of this trap (Figure 1). According to Agee *et al.* [19], *R. cerasi* has a major peak of electroretinographically-assessed spectral sensitivity at 485 to 500 nm (yellow green region) and a secondary peak at 365 nm (ultraviolet region). Trap Type F matches these specifications most closely of all tested trap types. 

Trap Types F and G were identical in color and material, apart from fluorescent pigments contained in trap Type G. No significant differences were observed between these two traps. Thus, fluorescence appears unnecessary for high *R. cerasi* captures. This result is contrary to literature references, which mention a positive effect of fluorescence on trap catches of *R. cerasi* [13,14]. However, for the western cherry fruit fly, *Rhagoletis indifferens*, highly fluorescent traps were found to be less attractive than traps with lower fluorescence [26].

Trap Types H and K were the least attractive. This observation is consistent with the literature: Prokopy and Boller [20] noted that pure yellow color with a sharp increase of reflectance between 500 to 520 nm is most attractive and that adding as little as 0.5% of blue or red enamel resulted in a significant decrease in fly capture. The reflectance patterns of the traps (Figure 1) show that trap Types H and K had an increased reflectance in the blue region (400–500 nm). 

**Figure 5:**
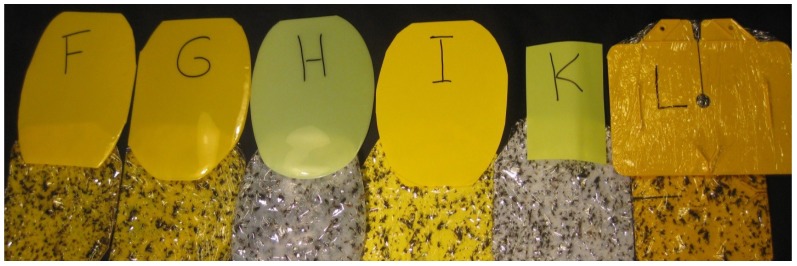
The color of trap types before and after the experiment (trap Type L = Rebell^®^ amarillo).

Captures of cherry fruit flies during the fifth week of the experiment (Figure 4, grey bars) were lower than during the second week of the experiment (Figure 4, white bars). This observation can be due to several reasons: (1) reduced fly abundance due to natural mortality; (2) reduced flight activity of the cherry fruit fly; (3) reduced attractiveness of the traps, due to fading of the yellow color; or (4) reduced attractiveness of the traps due to black pollution with already captured insects. The reduction of captures was most pronounced in trap Type K, which visually faded during the experimental period (Figure 5). Trap Type H also faded, but the reduction in captures was less pronounced. Both trap Types H and K were nearly white at the end of the experiment. Fading was less obvious in the other trap types. Trap Type I was a bit lighter at the end of the experiment and also showed a reduction in captures in the fifth week of the experiment. Trap Types F and G were very stable, because the yellow color was not printed onto the trap, but imbued within it. Reduced captures in trap Types F and G might also be due to pollution with already captured insects. Absolutely no visual fading was observed in the Rebell^®^ amarillo trap. This observation is in accordance with other experiments [27,28]. The Rebell^®^ amarillo trap contains highly-stable lead chromate yellow pigments and is intended to be reusable for several years after cleaning and new gluing. The Rebell^®^ amarillo trap showed the lowest reduction in captures. However, fading might have been of minor importance, because the rank order of captures of the different trap types was the same during the second and the fifth week of the experiment. Thus, we assume that the performance of the traps was stable over one flight period of *R. cerasi.* However, given the observed fading, the new trap types are not reusable in consecutive years. 

Trap Types I (polypropylene) and G (polyethylene) captured significantly more flies than trap Types K (polypropylene) and H (polyethylene). Thus, trap material was of minor importance compared to trap color.

**Figure 6:**
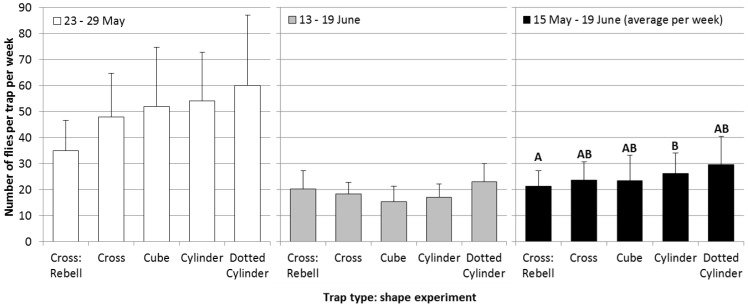
A comparison of differently-shaped trap types on the numbers of captured cherry fruit flies per week (+SE) during the second week of experiments (white bars), the fifth week of experiments (grey bars) and during the whole experimental period (black bars; average per week; Tukey’s HSD test *p* = 0.05, different letters show significant differences).

Experiment 2, comparison of different trap shapes: All traps in this experiment (Figure 2), except the standard Rebell^®^ amarillo trap, were made from the same material as trap Type I in the color experiment. Differences between trap shapes were not significant during the second and the fifth week of experiment (Figure 6; statistical analysis: data transformed [logx + 1], four-way-ANOVA, Tukey’s HSD-test; May 23–29: F_4,31 _= 0.8, *p* = 0.52; June 13–19: F_4,31 _= 1.8, *p* = 0.15). Over the whole experimental period, a significant difference was observed between the cylinder-shaped trap and the standard Rebell^®^ amarillo trap; this might have been due to differences in shape or differences in color (May 15–June 19: F_4,31 _= 3.1, *p* = 0.03). Apparently, shape is of minor importance as long as the object is three-dimensional and visible from all directions. 

The sticky surface was different among the tested trap types (Table 1): in the first experiment (color comparison), panels with a 600-cm^2^ surface were used. Trap types in the shape experiment had sticky surfaces of 900 cm^2^ (cube), 1080 cm^2^ (cylinder) and 1200 cm^2^ (cross), respectively. Although the cross-shaped traps had the largest sticky surface, captures were slightly lower than in cylinder-shaped traps. However, due to the shape, the outlines of the cylinder-shaped traps were the largest. In addition, the sticky surface in the inner angles of the cross-shaped traps does not catch a lot of flies, as it is difficult to reach.

Red dots on the cylinder increased total captures by 12%, but the differences were not significant. It is known that *Rhagoletis* flies react to red or dark-colored spheres of approximately the same size as the host fruit [18]. The attraction of fruit flies to spherical objects is believed to represent a response to mating and oviposition site-type stimuli. Red spheres of 7.5 cm in diameter are used as traps for the apple maggot fly, *Rhagoletis pomonella* [29]. The optimal sphere size for *R. cerasi* was determined to be 2.5 cm in diameter by Prokopy [12]. He also assumed that flies detect spheres solely on the basis of circular silhouette. Haisch and Forster [16], however, demonstrated that dots of 1 cm in diameter increased captures on yellow traps, whereas dots with larger diameters had no influence, and Russ *et al.* [30] showed that the combination of yellow surfaces with either cherry-sized discs or hemispheres did not significantly increase the attractiveness of traps. Observations by Wiesmann [31] indicate that flies detect the location of host fruit solely through vision. Dark-colored spheres are preferred, because they stand out in the strongest contrast against the background [17,32]. While fruit‑mimicking spherical traps are superior for attracting *R. pomonella*, yellow panels were found to be more suitable for capturing *R. cerasi* [12,33]. By adding the red dots to the yellow sticky trap, we tried to combine these two stimuli. However, further experiments on dot size, color and distribution are needed to improve trap efficiency.

## 4. Conclusions

Over the whole flight period, trap Types I and G were equally effective as the standard Rebell^®^ amarillo trap. Trap Type F was even significantly more effective than the Rebell^®^ amarillo trap. Thus, the new trap Type F is suitable for monitoring the beginning of the flight period, as well as for mass‑trapping purposes in home gardens. Color of trap Type F was stable during one flight period of cherry fruit fly. However, we assume that prolonged exposure to environmental conditions might lead to a desaturation of color. This might be a disadvantage compared to the standard Rebell^®^ amarillo trap, which contains highly-stable, but toxic, lead chromate yellow pigments and is intended to be reusable for several years after cleaning and new gluing. However, most farmers dispose the Rebell^®^ amarillo trap after one use, because cleaning the traps with solvents and re-gluing is a dirty, time-consuming task. The new trap type, made from thinner material and without lead chromate, might therefore be a good, eco-friendly alternative.

Trap shape seemed to be of minor importance. Based on economic and practical considerations, the cylinder shape seems to be the best option. This shape has several advantages: (1) it is easy to produce with a small amount of cutting loss, in contrast to the cube-shaped traps; (2) when laid out flat, it can be very economically packed and shipped; (3) the cylinder shape is stable, even if thin panels are used, unlike with cross-shaped traps; and (4) cylinder-shaped traps are easy for farmers to handle in the field. 

Red dots did not significantly increase captures. Further experiments are necessary to identify the optimal dot size and distribution. However, a commercial trap type without dots might be better, because: (1) the impact of red dots is considered to be rather low; (2) applying red dots would increase trap costs; and (3) because traps without dots can also be used for other pest insects (e.g., *Meligethes* sp*.* (Coleoptera: Nitidulidae) and *Ceutorhynchus* sp. (Coleoptera: Curculionidae) in oilseed rape production).

A commercial product “UFA-Samen Kirschenfliegenfalle” was developed based on these experiments: a cylinder-shaped trap (height 20 cm, circumference of 54 cm) made of Color F (yellow non-fluorescent polyethylene with a strong increase in reflectance at 500–550 nm, a secondary peak in the UV-region at 300–400 nm and a very low reflectance at ~450 nm). For cherry fruit fly monitoring—the detection of the presence/absence of pests and the detection of the first flight, to schedule precise insecticide applications—this product is now available and will be further evaluated under on-farm conditions in comparison with the standard Rebell® amarillo trap during the next few years. Further experiments are needed to investigate the efficacy of this new trap type for mass-trapping of *R. cerasi*.

## References

[1] Ranner H. (1988). Untersuchungen zur Biologie und Bekämpfung der Kirschfruchtfliege, *Rhagoletis cerasi* L. (Diptera, Trypetidae) - III. Statistischer Vergleich der Schlupfperioden und Schlupfraten der Kirschfliege. Pflanzenschutzberichte.

[2] Leski R. (1963). Studia nad biologia i ecologia nasionnicy tzresniowki *Rhagoletis cerasi* L. (Diptera: Trypetidae). Pol. Pismo Entomol. Ser. B.

[3] Boller E. (1966). Beitrag zur Kenntnis der Eiablage und Fertilität der Kirschenfliege *Rhagoletis cerasi* L. Mitt. Schweiz. Entomol. Ges..

[4] Wiesmann R. (1934). Untersuchungen über die Lebensgeschichte und Bekämpfung der Kirschenfliege *Rhagoletis cerasi* Linné - II. Mitteilung. Landw. Jahrb. Schweiz..

[5] Thiem H. (1934). Beiträge zur Epidemiologie und Bekämpfung der Kirschfruchtfliege (*Rhagoletis cerasi* L.). Arb. Physiol. Angew. Entomol. Berl. Dahlem..

[6] Fimiani P., Cavalloro R. (1983). Multilarval infestations by *Rhagoletis cerasi* L. (Diptera: Trypetidae) in cherry fruits. Fruit flies of economic importance.

[7] Daniel C., Grunder J. (2012). Integrated Management of European Cherry Fruit Fly *Rhagoletis cerasi* (L.): Situation in Switzerland and Europe. Insects.

[8] Daniel C., Wyss E. (2010). Field applications of *Beauveria bassiana* to control the European Cherry Fruit Fly *Rhagoletis cerasi*. J. Appl. Entomol..

[9] Böckmann E., Hummel E., Vogt H. Promising field and semi field results for cherry fruit fly control using neem. Proceedings  of the 15th International Conference on Organic Fruit-Growing.

[10] Böckmann E., Köppler K., Hummel E., Vogt H. (2014). Bait spray for control of European cherry fruit fly—An appraisal based on semi-field and field studies. Pest Manag. Sci..

[11] Thistlewood H., Bostanian N., Senger S., DeLury N. (2010). Adapting to new management strategies for cherry fruit flies in British Columbia, Canada. IOBC/WPRS Bull..

[12] Prokopy R.J. (1969). Visual responses of European cherry fruit flies—*Rhagoletis cerasi* L. (Diptera, Trypetidae). Pol. Pismo Entomol..

[13] Boller E. (1969). Neues über die Kirschenfliege: Freilandversuche im Jahr 1969. Schweiz. Z. Obst-und Weinbau.

[14] Remund U. (1971). Anwendungsmöglichkeiten einer wirksamen visuellen Wegwerffalle für die Kirschenfliege. Schweiz. Z. Obst-und Weinbau.

[15] Haisch A., Forster S. (1969). Versuche zur Anköderung und zum Fang der Kirschenfliege (*Rhagoletis cerasi* L.). Anz. Schadl. J. Pest Sci..

[16] Haisch A., Forster S. (1970). Erfahrungen beim Fang der Kirschenfliege mit Leimtafeln und Leimkugeln. Gesunde Pflanz..

[17] Katsoyannos B.I., Robinson A.S., Hooper G. (1989). Response to shape, size and color. Fruit flies Their Biology, Natural Enemies and Control.

[18] Prokopy R.J. (1971). Orientation of the apple maggot flies *Rhagoletis pomonella* (Walsh) and European cherry fruit flies *R. cerasi* L. (Diptera: Tephritidae) to visual stimuli. Proceedings of the 13 International Congress of Entomology.

[19] Agee H.R., Boller E., Remund U., Davis J.C., Chambers D.L. (1982). Spectral sensitivities and visual attractant studies on the Mediterranean Fruit Fly, *Ceratitis capitata* (Wiedemann), Olive Fly, *Dacus oleae* (Gmelin), and the European Cherry Fruit Fly, *Rhagoletis cerasi* (L) (Diptera, Tephritidae). J. Appl. Entomol..

[20] Prokopy R.J., Boller E. (1971). Response of European Cherry Fruit Flies to colored rectangles. J. Econ. Entomol..

[21] Remund U., Boller E. (1978). Kirschenfliegenfallen für Prognosewesen und biotechnische Bekämpfung im Vormarsch. Schweiz. Z. Obst-und Weinbau.

[22] Dayan A.D., Pajne A.J. (2001). Mechanisms of chromium toxicity, carcinogenicity and allergenicity: Review of literature from 1985 to 2000. Hum. Exp. Toxicol..

[B23-insects-05-00564] 23.Regulation (EC) No 1907/2006 of the European Parliament and of the Council concerning the Registration, Evaluation, Authorisation and Restriction of Chemicals (REACH), establishing a European Chemicals Agency, amending Directive 1999/45/EC of the European Parliament and of the Council and repealing Council Regulation (EEC) No 793/93 and Commission Regulation (EC) No 1488/94, as well as Council Directive 76/769/EEC and Commission Directives 91/155/EEC, 93/67/EEC, 93/105/EC and 2000/21/EC.

[24] Samietz J., Graf B., Höhn H., Schaub L., Höpli H.U. (2007). Phenology modelling of major insect pests in fruit orchards from biological basics to decision support: the forecasting tool SOPRA. EPPO Bull..

[25] (2003). JMP.

[26] Yee W.L. (2013). Preference by *Rhagoletis indifference* (Diptera, Tephritidae) for rectangles of various yellow colours and fluorescence. J. Appl. Entomol..

[27] Liburd O.E., Stelinsky L.L., Gut L.J., Thornton G. (2001). Performance of various trap types for monitoring populations of Cherry Fruit fly (Diptera: Tephritidae) species. Environ. Entomol..

[28] Katsoyannos B.I., Papadopoulos N.T., Stavridis D. (2000). Evaluation of trap types and food attractants for *Rhagoletis cerasi* (Diptera: Tephritidae). J. Econ. Entomol..

[29] Prokopy R.J. (1968). Visual responses of Apple Maggot Flies, *Rhagoletis pomonella* (Diptera: Tephritidae): Orchard studies. Entomol. Exp. Appl..

[30] Russ K., Boller E., Vallo V., Haisch A., Sezer S. (1973). Developement and application of visual traps for monitoring and control of populations of *Rhagoletis cerasi* L. Entomophaga.

[31] Wiesmann R. (1937). Die Orientierung der Kirschfliege, *Rhagoletis cerasi* L., bei der Eiablage. Landw. Jahrb. Schweiz..

[32] Levinson H.Z., Haisch A., Cavalloro R. (1983). Optical and chemosensory stimuli involved in host recognition and oviposition of the cherry fruit fly *Rhagoletis cerasi* L. Fruit flies of economic importance.

[33] Economopoulos A.P., Robinson A.S., Hooper G. (1989). Use of traps based on color and/or shape. Fruit flies their biology, natural enemies and control.

